# Re-interpreting conventional interval estimates taking into account bias and extra-variation

**DOI:** 10.1186/1471-2288-6-51

**Published:** 2006-10-16

**Authors:** Michael Höfler, Shaun R Seaman

**Affiliations:** 1 Institute of Clinical Psychology and Psychotherapy, Dresden University of Technology, Chemnitzer Str. 46a, 01187 Dresden, Germany; 2 Department of Statistical Science, University College London, Gower Street, London WC1E 6BT, UK

## Abstract

**Background:**

The study design with the smallest bias for causal inference is a perfect randomized clinical trial. Since this design is often not feasible in epidemiologic studies, an important challenge is to model bias properly and take random and systematic variation properly into account. A value for a target parameter might be said to be "incompatible" with the data (under the model used) if the parameter's confidence interval excludes it. However, this "incompatibility" may be due to bias and/or extra-variation.

**Discussion:**

We propose the following way of re-interpreting conventional results. Given a specified focal value for a target parameter (typically the null value, but possibly a non-null value like that representing a twofold risk), the difference between the focal value and the nearest boundary of the confidence interval for the parameter is calculated. This represents the maximum correction of the interval boundary, for bias and extra-variation, that would still leave the focal value outside the interval, so that the focal value remained "incompatible" with the data. We describe a short example application concerning a meta analysis of air versus pure oxygen resuscitation treatment in newborn infants. Some general guidelines are provided for how to assess the probability that the appropriate correction for a particular study would be greater than this maximum (e.g. using knowledge of the general effects of bias and extra-variation from published bias-adjusted results).

**Summary:**

Although this approach does not yet provide a method, because the latter probability can not be objectively assessed, this paper aims to stimulate the re-interpretation of conventional confidence intervals, and more and better studies of the effects of different biases.

## Background

Conventional causal estimates from observational data involve many assumptions, e.g. assumptions about random exposure assignment, selection and participation, ignorable missing data and absence of measurement error [[1], *ch. 12–17*; [2,3]]. Although causal systems in epidemiology are commonly assumed to be so complex that one cannot expect to understand or correct for all biases, one can hope to adjust for the major ones and estimate uncertainty more accurately [2].

Conventional frequentist analyses often yield biased point estimates because they implicitly set all bias parameters (e.g. misclassification probabilities) to zero. Bias also arises from misspecation of models, e.g. the ignoring of covariates or of heterogeneity in individual effects [4,5]. Moreover, the interval estimates in conventional analyses reflect at most random errors and not systematic errors (biases). Ignoring uncertainty about bias parameters can make the intervals too narrow [2,6,7]. Importantly, while random error decreases with increasing sample size, uncertainty about biases remains [2,6,7]. Random error due to sampling and randomisation is often modelled in an unrealistically simple way. Usual regression, ANOVA and ANCOVA models, for instance, assume that the outcome has equal means and variances after conditioning on the covariates. However, unobserved covariates and varying individual effects may cause heterogeneity in the outcome's mean and variance structures [8-12]. These problems can often be addressed by the use of appropriate mixed and multi-level models [5]. Furthermore, exploratory analyses that precede the final analysis are often ignored when modelling random error. These include simplifying a regression model (e.g. omitting nonsignificant main-effect and interaction terms) and categorisation of continuous variables [13]. Finally, design issues such as the presence of clustered observations or multi-stage sampling [14] are often ignored but can also be considered in multi-level models [5].

In this paper we use the term "extra-variation" to summarize uncertainties about biases and unmodelled random error. The term "bias" denotes all biases and is defined as the difference between the expectation of the estimator and the true average causal effect. Strictly speaking, we mean "expected bias" because we invoke a Bayesian model that involves priors.

There are basically five approaches to address bias and uncertainties about bias (some of which also address unmodelled random-error):

1. Most frequently, only intuitive discussions about the impact of bias are provided. These are often found to be wrong when evaluated quantitatively, for instance, when misclassification is falsely assumed to be non-differential for all individuals [15,16].

2. Sometimes, a subsample is used to investigate a single source of bias (most often measurement error). Such validation studies, however, are often small, giving large random error, and may also be biased, e.g. due to selection bias [17].

3. In sensitivity analysis several bias scenarios are investigated. Bias parameter values are added to the model and values specified for these. The data are then analysed supposing these values to be true, and the dependence of the results on the assumed values of these unknown bias parameters examined. However, sensitivity analysis often shows only that a great variety of results are possible if bias parameters are chosen accordingly.

4. In Monte Carlo sensitivity analysis (MCSA), distributions for the bias parameters are specified. Causal-effect estimates are then sampled by drawing bias parameter values from their distributions and repeating the analysis for each draw [6].

5. Bayesian methods complete the hierarchy of sophistication. Here, the posterior distribution of the causal effect is calculated given the data and the priors for the bias parameters. Uncertainties about the unknown parameters are incorporated through prior distributions. These may be derived from other data sources such as validation studies. MCSA results have an approximate Bayesian interpretation if the estimator of the causal effect is efficient, the data are uninformative about the bias parameters and the MCSA procedure is modified by adding normal disturbances to the causal-effect estimates [2]. From the Bayesian perspective, frequentist confidence intervals are Bayesian intervals with inappropriate point priors at zero for bias parameters, e.g. misclassification probabilities [2,7], and flat priors for the causal effect. Priors at zero for the bias parameters are inappropriate if the data are not randomly sampled or not randomly assigned to groups [18]. The use of flat priors for causal effects can be criticised, as it implies that a risk ratio of, say, 10^-5^, is a priori as probable as a risk ratio of 1.5 [19]. Nevertheless, in practice, such priors are often used in Bayesian analyses.

Instead of modifying the interval estimate, as is done in all the quantitative approaches mentioned above, we propose to re-interpret it. The general procedure is as follows:

1. Suppose we are interested in whether a particular value (called the focal value) of a parameter of interest is "compatible" with the observed data. The parameter could be, for example, the risk ratio (RR) and the focal value might be 1, which is its null value, or it could be some non-null value, e.g. 2. Suppose this focal value lies outside the frequentist confidence interval (CI), so that we say it is "incompatible" with the data. (Although, of course, in reality even a perfect interval estimate would exclude nothing with certainty [20].) This "incompatibility" may, however, be due to bias or extra-variation.

2. Calculate the difference between the focal value and the nearest boundary of the CI. This difference represents the maximum permitted correction (MPC) to the interval boundary that would still leave the interval excluding the focal value.

3. Sources of possible bias and extra-variation in the study would then be examined to assess how likely it is that the appropriate correction is less than the MPC.

This procedure aims to improve intuitive discussions on bias by assessing the probability in stage 3. After presenting a motivational example, we derive the maximum permitted correction in the general case and for risk ratios and risk differences, and describe an application to a meta analysis of air versus oxygen resuscitation treatment in newborn infants. Finally, we present some general guidelines for the assessment of the probability in 3.

## Discussion

### Motivational example

Suppose we are assessing the average causal effect of a binary exposure *X *on a binary outcome *Y*, quantified by the risk ratio (RR). If the outcome is rare under all exposure and covariate levels, the odds ratio (OR) approximates the RR.

Asssume that in a study of a rare disease 200 of 2000 undiseased and 20 of 120 diseased individuals are exposed. The OR is (2000-200)*20/{200*(120-20)} = 1.8. An estimate of the standard error (SE) of the natural logarithm (log) OR is (1/1800 + 1/200 + 1/100 + 1/20)^1/2 ^= 0.256. Using the Wald method, the 95% CI for log OR is log(1.8) +/- 1.96 * 0.256 = 0.086 – 1.090. Thus, the correction of the lower boundary of the interval must be smaller than 0.086 if the interval is to exclude 0.

Now, suppose for illustration that the only bias is due to misclassification in the disease status which operates in such a way that some exposed individuals without disease may be classified as diseased. Were 3 of the 20 apparently diseased exposed individuals actually to be undiseased, the OR would decrease to (1800*17)/(100*203) = 1.51 and the lower limit of the confidence interval would be log(1.51)-1.96*(1/1800 + 1/203 + 1/100 + 1/17)^1/2 ^= -0.052. The CI now includes zero. Hence, the null value of the log OR, 0, excluded by the original CI, would then lie within the interval. Thus, if it is likely that at least 3 of the 20 apparently diseased exposed individuals have been misclassified, it is likely that the shift required in the CI boundary is more than the maximum permitted correction: the null value would then be compatible with the data. Note that the simple calculation above did not address uncertainty in the misclassification probability, a probability which might have been estimated from a validation dataset. Methods for taking in account such uncertainty are described in [21].

### The maximum permitted correction

The average causal effect of *X *= 1 versus *X *= 0 on an outcome *Y *(not necessarily binary) could be assessed using different indices depending on the outcome, the study design and the research aim. The index could be a multiplicative measure like RR or OR, or an additive measure like the risk difference (RD). For differences "average effect" refers to the arithmetic mean of the individual effects, whereas for ratios it refers to the geometric mean. (Note that the odds ratio can only be interpreted in this way if the vast majority of individual risks are low [22].) Let *θ *denote the parameter to be estimated; i.e., the population-average effect for additive measures or the log-population average effect for multiplicative measures. (One could also use other smooth functions of the mean causal effect, but the population-average interpretation might then fail.)

Let θ^obs
 MathType@MTEF@5@5@+=feaafiart1ev1aaatCvAUfKttLearuWrP9MDH5MBPbIqV92AaeXatLxBI9gBaebbnrfifHhDYfgasaacH8akY=wiFfYdH8Gipec8Eeeu0xXdbba9frFj0=OqFfea0dXdd9vqai=hGuQ8kuc9pgc9s8qqaq=dirpe0xb9q8qiLsFr0=vr0=vr0dc8meaabaqaciaacaGaaeqabaqabeGadaaakeaaiiGacuWF4oqCgaqcamaaBaaaleaacqWGVbWBcqWGIbGycqWGZbWCaeqaaaaa@32C8@ denote the model-based point estimate of *θ *from a frequentist analysis, and (*l*_*obs*_, *u*_*obs*_) be the (1-α)*100 % CI. Bias may have been reduced by adjusting for observed confounders and/or by using weights to account for known selection bias [23]. If *θ *is the log OR, θ^obs
 MathType@MTEF@5@5@+=feaafiart1ev1aaatCvAUfKttLearuWrP9MDH5MBPbIqV92AaeXatLxBI9gBaebbnrfifHhDYfgasaacH8akY=wiFfYdH8Gipec8Eeeu0xXdbba9frFj0=OqFfea0dXdd9vqai=hGuQ8kuc9pgc9s8qqaq=dirpe0xb9q8qiLsFr0=vr0=vr0dc8meaabaqaciaacaGaaeqabaqabeGadaaakeaaiiGacuWF4oqCgaqcamaaBaaaleaacqWGVbWBcqWGIbGycqWGZbWCaeqaaaaa@32C8@ can be computed by logistic regression; if a log-rate ratio, by Poisson regression.

Imagine a hypothetical Bayesian multiple bias model that removes all biases completely and take all uncertainties about bias parameters and random error perfectly into account. As in Greenland [2], we assume a non-informative prior for the causal effect. Let (*l*_*perf*_, *u*_*perf*_) denote the associated interval estimate from this model; i.e., the α/2 and 1-α/2 quantiles of the Bayesian posterior distribution of *θ *given the data and the hypothetical perfect bias model. Discrepancies between (*l*_*obs*_*, u*_*obs*_) and (*l*_*perf*_, *u*_*perf*_) are due to biases, uncertainties about biases and unmodelled random variation.

Let *a *and *b *be the shifts in the interval boundaries, i.e.:

*l*_*perf *_= *l*_*obs *_- *a*

and

*u*_*perf *_= *u*_*obs *_+ *b*.

#### The null value as focal value

Suppose the focal value is 0, and that the interval [*l*_*obs*_*, u*_*obs *_] excludes 0. If *l*_*obs *_> 0, the MPC (in the lower boundary) simply equals *l*_*obs*_, as we require that *l*_*obs*_*- a *> 0. This result is often applied intuitively: it simply indicates that, the further the lower boundary is from the null, the more room there is for bias and extra-variation. If *u*_*obs *_< 0, the MPC (in the upper boundary) equals *u*_*obs*_.

#### Other focal values

Alternatively, one may require that the causal effect, *θ*, exceeds some pre-specified non-null focal value, *κ*, that reflects a relevant threshold for clinical or policy significance. Assuming *l*_*obs*_*> κ*, the MPC is *l*_*obs *_- *κ*. Likewise, one may ask whether *θ *is smaller than a certain *κ *which corresponds to little harm. Assuming *u*_*obs*_*< κ*, the MPC is *κ *- *u*_*obs*_.

### Special cases: RR, RD and number needed to treat

If *θ *= log RR, one is often interested in effects of at least a *q*-fold risk (e.g. *q *= 2). Here, *κ *= log(*q*), so that *a *must be less than *l*_*obs *_- log(*q*).

If *θ *is RD, then demonstrating a RD of more than *κ *(e.g. *κ *= 0.1) requires that *a *be less than *l*_*obs *_- *κ*. RD equals the inverse number needed to treat (NNT), the number of individuals required to prevent (or delay) one adverse event. Although NNT has often been misinterpreted [24], this measure is becoming increasingly prominent in clinical epidemiology because of its intuitive meaning [25]. Showing that NNT <*q *is equivalent to specifying that RD > *κ *= 1/*q*.

### Assessing whether the maximum permitted correction is sufficient

We need to estimate the probability that the true shift, *a *or *b*, is less than the maximum correction that would leave the focal value "incompatible" with the data. This probability should be assessed with respect to understood bias. Of course, the result could still be distorted by non-understood or unknown bias. First, one should assess the shift due to bias by looking at studies that have investigated specific biases or global bias. Second, a further correction should be added for the uncertainty about the bias parameter values. The magnitude of this correction depends on the size of the studies from which bias was estimated, the uncertainty about their applicability to the present data and assumptions made about bias in these studies. Third, a correction due to extra random variation should be added. The magnitude of the true shift could be estimated from studies (simulations or real-data studies) that have compared, in similar settings, naive interval estimates with estimates obtained using more sophisticated methods. Finally, one would compare the shift, *a *or *b*, estimated using the above procedure with the MPC for the focal value.

This approach aims to improve the intuitive assessment of bias by relating it to MCSA and Bayesian methods. These procedures allow *a *and *b *to be estimated based on understood bias. Knowledge of such analyses should enable researchers to improve their assessments about *a *and *b*. In the discussion we give more guidance for assessing these shifts.

### Application

Davis et al. performed a meta analysis of five clinical trials of 100 % oxygen versus air resuscitation treatment of newborns [26]. Resuscitation treatment aims to prevent death and longterm adverse neurodevelopmental consequences in newborns with breathing difficulties ("asphyxia"). Although oxygen has been recommended for many years, some researchers are concerned about possible side-effects of pure oxygen on cerebral blood flow and the generation of oxygen free radicals (see [26], and references therein). We focus here on the core outcome "death at latest follow-up" (death during the first week in three studies and death during the first four weeks in one study; in the remaining study it was not assessed, so we exclude this study).

Of the four trials, one was randomized and the care-providers and outcome-assessors were blind to treatment status. The other three studies were only quasi-randomized and without blinding. Three studies allowed backup-therapy with oxygen therapy if air therapy was unsuccessful and one study excluded the individuals who received backup therapy. Davis et al [26] used a fixed-effects model and found a higher death rate among newborns with oxygen resuscitation: 107 out of 659 individuals treated with oxygen died versus 70 out of 616 with air; the RD, θ^obs
 MathType@MTEF@5@5@+=feaafiart1ev1aaatCvAUfKttLearuWrP9MDH5MBPbIqV92AaeXatLxBI9gBaebbnrfifHhDYfgasaacH8akY=wiFfYdH8Gipec8Eeeu0xXdbba9frFj0=OqFfea0dXdd9vqai=hGuQ8kuc9pgc9s8qqaq=dirpe0xb9q8qiLsFr0=vr0=vr0dc8meaabaqaciaacaGaaeqabaqabeGadaaakeaaiiGacuWF4oqCgaqcamaaBaaaleaacqWGVbWBcqWGIbGycqWGZbWCaeqaaaaa@32C8@, equals 0.05, 95 % CI is 0.01 – 0.08. No more decimal places are provided, but in favour of a stronger effect, we assume a lower CI boundary of 0.014 for the following calculations. Thus, for a focal value of zero, i.e. no effect, the MPC is 0.014.

Davis et al interpret their results as evidence to prefer initial use of air resusciation and to use oxygen as backup if necessary. This is a conclusion about clinical practice: about which treatment to use first given that the other treatment may be available as back-up. If one is interested in the pure efficacy of the competing treatments, i.e., how well they work when used alone, then in the absence of other biases, the RD estimate is likely to be biased towards no effect (i.e. underestimated) because of the availability of back-up oxygen treatment in three studies. The lack of blinding in three of the four studies could have caused over-estimation of RD, as has been found in other studies [27]. Publication bias may suggest over-estimated effects because small (under-powered) studies with non-significant results may be less likely to be published [28-31], or to under-estimation due to industry suppressing adverse findings (although the latter seems unlikely in this case). Other potential causes of bias include the quasi-randomisation in some of the studies and variation in follow-up duration. Because of the small study sizes and the small number of studies, the impacts of these design issues cannot be well estimated.

There are various likely sources of extra-variation in the estimated risk difference. First, there is the uncertainty in the bias parameters: the results of Davis et al. assume these are zero. Second, there is unmodelled random variation, e.g. due to heterogeneity in effect magnitude between the studies. Heterogeneity was not statistically significant but this may be due to low power, and it is known that fixed-effect models underestimate the standard error when there is heterogeneity [32]. Unmodelled random variation could also have arisen from shared, unobserved factors at the level of clinicians or trials which induced correlation in observations, or from unmodelled individual heterogeneity in treatment response (which might vary e.g. according to pulmonary hypertension, as mentioned by the authors).

In conclusion, there could be several sources of bias and extra-variation in the results. Information about the magnitude of the bias parameters appears sparse, so large uncertainty about them should be allowed, as well as considerable unmodelled random error. It seems likely that the true, unknown shift, *a*, is greater than 0.014: no clear preference for either treatment can be inferred.

## Summary

The approach advocated in this paper involves the assessment of the probability that the true shift in the relevant confidence bound is less than the maximum permitted correction. Even for experts this is a very difficult task and experts might disagree substantially in their assessment. From cognitive psychology it is known since the 1970s that people tend to rely on a small number of simple heuristics when faced with decisions based on probability assessments [33-36]. The reliance on such simple heuristics, however, can be very prone to errors [33-35]. Even statistically educated psychologists were found to make severe errors when assessing probabilities [36]. More than two decades later, however, Gigerenzer and his group showed that people do indeed tend to use simple heuristics, but that they are right in many instances (summarized in [37]). They explained the earlier results by demonstrating that contextual information (e.g. wording of questions) plays an important role in determining the answer given, and that humans can be led systematically to give a "false" answer. Bearing all this in mind, here are some conceptional guidelines to the problem:

1. The probability that the maximum permitted correction is sufficient should be specified according to *understood *bias. The inference could still be distorted by misunderstood or unknown bias, as well as by residual uncertainty.

2. We recommend using information from similar studies or MCSA and Bayesian analyses applied in similar situations. The results from such studies can be used as a crude basis for the assessment of the probability that the MPC is sufficient. In cognitive psychology, information used for an assessment that is taken from similar entities or settings is called "*anchor *information". In many applications, specific biases have been investigated, for instance, by comparing an instrument with small measurement error with another known to have much larger error. However, such estimates of specific biases and associated uncertainty are themselves error-prone because of potential incomparability of studies with respect to other biases (and random error). Moreover, the same kind of bias might operate differently in different studies. For instance, an instrument with good validity in clinical populations might perform poorly in general populations. Therefore using information from other studies has to be done very carefully because it could cause more harm than good.

Likewise, cognitive psychology tells us that anchor information can be quite misleading [37-42] and that almost anything could serve as an anchor when a subject is faced with the task of estimation. Therefore, strategies are required to separate useful from useless or even misleading anchor information and to take into account uncertainty about their applicability. Moreover, there are various ways of combining different kinds of bias when assessing global bias and global unmodelled variance. The easiest is just to sum them up, but they may act dependently on one another. The less anchor information there is and the less precise is that information, the more the boundary should be shifted, in addition to the shift due to assumed bias.

3. The shift in the interval boundaries could be directly estimated by MCSA or Bayesian methods. However, these analyses are not easy for non-experts to conduct. We expect, on the other hand, that the more results researchers see from such analyses, the more they would develop an intuitive feeling for the effects that multiple bias and extra variation might have in specific situations. At the very least, such analyses show that one should have much less confident in conventional analyses than is suggested by their confidence intervals.

However, given these guidelines there remains much uncertainty and subjectivity in assessing the probability that the MPC is sufficient. Therefore, the way of re-interpreting confidence intervals described in this paper does not yet constitute a method. Further studies on bias are required to provide more objective information and to render the approach more useful. This paper is intended to be just a starting point for thinking about re-interpreting conventional confidence limits.

## Abbreviations

MCSA = Monte Carlo sensitivity analysis

CI = confidence interval

RR = risk ratio

OR = odds ratio

log = natural logarithm

RD = risk difference

NNT = number needed to treat

MPC = maximum permitted correction

## Competing interests

The author(s) declare that they have no competing interests.

## Authors' contributions

Both MH and SRS have contributed to all aspects of the content.

## Pre-publication history

The pre-publication history for this paper can be accessed here:



## References

[B1] Rothman KJ, Greenland S (1998). Modern Epidemiology.

[B2] Greenland S (2005). Multiple-bias modelling for analysis of observational data. J Roy Stat Soc, Series A.

[B3] Maclure M, Schneeweiß S (2001). Causation of bias: the episcope. Epidemiol.

[B4] Neuhaus JM, Hauck WW, Kalbfleisch JD (1992). The effects of mixture distributions misspecification when fitting mixed-effects logistic models. Biometrika.

[B5] Skrondal A, Rabe-Hesketh S (2004). Generalized latent variable modeling.

[B6] Greenland S (2001). Sensitivity analysis, Monte Carlo risk analysis and Bayesian uncertainty assessment. Risk Anal.

[B7] Greenland S (2004). Interval estimation by simulation as an alternative to and extension of confidence intervals. Int J Epidem.

[B8] Gray G (1994). Bias in misspecified models. Biometrics.

[B9] Harville DA, Mee RW (1984). A mixed-model procedure for analysing ordered categorical data. Biometrics.

[B10] Liang K-Y, McCullagh P (1993). Case studies in binary dispersion. Biometrics.

[B11] Kachman SD, Everett RW (1993). A multiplicative mixed model when the variances are heterogeneous. J Dairy Sci.

[B12] Greenland S (2000). When should epidemiologists use random coefficients?. Biometrics.

[B13] Begg MD, Lagakos S (1990). On the consequences of model misspecification in logistic regression. Environm Health Perspect.

[B14] Levy PS, Lemeshow S (1999). Sampling of populations.

[B15] Jurek AM, Greenland S, Maldonado G, Church TR (2005). Proper interpretations of non-differential classification effects: expectations vs observations. Int J Epidemiol.

[B16] Höfler M (2005). The effect of misclassification on the estimation of association: a review. Int J Meth Psychiat Res.

[B17] Rosner B, Gore G (2001). Measurement error correction in nutritional epidemiology based on individual foods, with application to the relation of diet to breast cancer. Amer J Epidemiol.

[B18] Greenland S (1990). Randomization, statistics, and causal inference. Epidemiology.

[B19] Greenland S (2006). Bayesian perspectives for epidemiologic research. Int J Epidemiol.

[B20] Poole C (1987). Confidence intervals exclude nothing. Am J Public Health.

[B21] Gustafson P (2004). Measurement error and misclassification in statistics and epidemiology.

[B22] Greenland S (1987). Interpretation and choice of effect measures in epidemiological analyses. Amer J Epidem.

[B23] Höfler M (2005). The use of weights to account for non-response and drop-out. Soc Psychiat & Psychiat Epidemiol.

[B24] Christensen PM, Kristiansen IS (2006). Number-needed-to-treat (NNT) – Needs treatment with care. Basic & Clin Pharmacol & Toxicol.

[B25] Kristiansen IS, Gyrd-Hansen D, Nexoe J, Nielsen JB (2002). Number needed to treat: easily understood and intuitively meaningful? Theoretical considerations and a randomized trial. J Clin Epidemiol.

[B26] Davis PG, Tan A, Schulze A (2004). Resuscitation of newborn infants with 100 % oxygen or air: a systematic review and meta analysis. Lancet.

[B27] Schulz KF, Chalmers I, Hayes RJ, Altman DG (1995). Empirical evidence of bias. Dimensions of methodological quality associated with estimates of treatment effects in controlled trials. J Am Med Ass.

[B28] Newcombe RG (1987). Towards a reduction in publication bias. Brit Med J.

[B29] Gilbody SM, Song F, Eastwood AJ, Sutton A (2000). The causes, consequences and detection of publication bias in psychiatry. Acta Psychiat Scand.

[B30] Thornton A, Lee P (2000). Publication bias in meta-analysis: its causes and consequences. J Clin Epidemiol.

[B31] Sutton AJ, Duval SJ, Tweedie RL, Abrams KR, Jones DR (2000). Empirical assessment of effect of publication bias on meta-analyses. Brit Med J.

[B32] Poole C, Greenland S (1999). Random-effects meta-analyses are not always conservative. Am J Epidemiol.

[B33] Kahnemann D, Tversky A (1973). On the psychology of prediction. Psychol Rev.

[B34] Tversky A, Kahnemann D (1974). Judgment under uncertainty: heuristics and biases. Science.

[B35] Tversky A, Slovic P, Kahnemann D, (eds) (1982). Judgment under uncertainty: heuristics and biases.

[B36] Tversky A, Kahneman D (1971). Belief in the law of small numbers. Psychol Bull.

[B37] Gigerenzer G, Todd PM, the ABC research group (1999). Simple heuristics that make us smart.

[B38] Parducci A, Perrett DS, Marsh HW (1969). Assimilation and contrasts as range-frequency effects of anchors. J Exper Psychol.

[B39] Murphy KR, Constans JI (1987). Behavioral anchors as a source of bias in rating. J Appl Psychol.

[B40] Montgomery RL (1980). Reference groups as anchors in judgments of other groups: a biasing factor in "rating tasks". Psychol Reports.

[B41] Wedell DH, Parducci A, Lane M (1990). Reducing the dependence of clinical judgment on the immediate context: effects of number of categories and type of anchors. J Personal and Soc Psychol.

[B42] Epley N, Gilovich T (2001). Putting adjustment back in the anchoring and adjustment heuristic: Differential processing of self-generated and experimenter-provided anchors. Psychol Science.

